# Supplementary material for: Direct imaging of magnetohydrodynamic wave mode conversion near a 3D null point on the sun
Source: Nat Commun. 2024 Mar 26;15:2667. doi: 10.1038/s41467-024-46736-4 (PMC10965924; doi:10.1038/s41467-024-46736-4)
Supplement: Supplementary file 2 — Description of Additional Supplementary Files [file 41467_2024_46736_MOESM2_ESM.pdf]

Description of Additional Supplementary Files

File name: Supplementary Movie 1
Description: STEREO EUVI-B 195 Å intensity and running difference movie showing the flare, eruption, and the associated fast EUV wave.

File name: Supplementary Movie 2
Description: SDO/AIA 193 Å intensity and running difference movie showing the propagation of fast EUV wave through the null and appearance of slow EUV waves.

File name: Supplementary Movie 3
Description: SDO/AIA 193 Å running difference movie showing the propagation of fast EUV wave along slice SL1.

File name: Supplementary Movie 4
Description: SDO/AIA 193 Å running difference movie showing the propagation of slow EUV waves along slice SL2.

File name: Supplementary Movie 5
Description: SDO/AIA 171 Å intensity movie showing the decaying kink oscillations along slices PQ and RS.
